# ALDH3A1 driving tumor metastasis is mediated by p53/BAG1 in lung adenocarcinoma

**DOI:** 10.7150/jca.58250

**Published:** 2021-06-11

**Authors:** Feifei Fan, Ruxue Yin, Liuya Wang, Shunxin Zhao, Dan Lv, Kangli Yang, Shen Geng, Ningning Yang, Xiaohong Zhang, Hongmin Wang

**Affiliations:** 1 Department of Respiratory Medicine, The First Affiliated Hospital of Zhengzhou University, Zhengzhou 450052, China.; 2 Department of rheumatism and immunology, The First Affiliated Hospital of Zhengzhou University, Zhengzhou 450052, China.; 3 The First Affiliated Hospital of Zhengzhou University, Zhengzhou 450052, China.; 4 Department of Respiratory Medicine, Zhengzhou Central Hospital, Zhengzhou 450052, China.

**Keywords:** lung adenocarcinoma, metastasis, ALDH3A1, proliferation, prognosis

## Abstract

Lung adenocarcinoma (LUAD) is a lethal malignancy with metastasis, a major tumor feature that predominantly correlated with progression, but the molecules that mediated tumor metastasis remain elusive. To declare the critical regulatory genes, RNA sequencing data in LUAD patients was acquired from The Cancer Genome Atlas (TCGA) and found that ALDH3A1 was distinctly highly expressed in LUAD patients with metastasis (M1) compared with those without metastasis (M0), linked to the property of cancer stem cell and epithelial-mesenchymal transition (EMT). Besides, high ALDH3A1 expression predicted a poor prognosis. Knockdown of ALDH3A1 showed decreased proliferation, migration, and invasion in A549 cell line. Furthermore, BAG1 was regulated by ALDH3A1 through p53, enhanced cell proliferation, and predicted clinical prognosis. Our findings collectively uncovered a novel mechanism that orchestrates tumor cells' metastasis, and decreasing ALDH3A1 represented a potential therapeutic target for reprogramming metastasis.

## Introduction

Tumor recurrence and metastasis are the leading cause of cancer-related mortality 1. Lung adenocarcinoma (LUAD), belonging to a kind of non-small cell lung cancer, is often detected with metastasis in bones, brain, and respiratory system, thereby permitting metastasis-mediated clinical relapse 2. Although novel tumor therapies are rapidly developed, surgery and chemotherapy/radiotherapy are still the current standard treatment options for most LUAD patients, without improving recurrence and metastasis of LUAD 3. Therefore, a clear understanding of underlying mechanisms of tumor development and metastasis is required to discover novel therapeutic targets in LUAD.

Aldehyde dehydrogenase 3A1 (ALDH3A1), a member of NAD+ dependent enzymes, oxidizes diverse endogenous and exogenous aldehydes to carboxylic acids 4. In human corneal epithelial cells, Voulgaridou GP and co-authors demonstrated that ALDH3A1 protected corneal epithelial cells from oxidation by maintaining DNA integrity 5. Since ALDH3A1 plays a critical role in normal cells, efforts to understanding this role in cancers have largely been reported. For instance, ALDH3A1 expression was required for cell viability in esophageal squamous cell carcinoma cell lines 6. ALDH3A1 was found in lung cancer with abnormal expression, correlated with histologic type 7. Besides, Sullivan JP et al. reported that aldehyde dehydrogenase could be known as a candidate marker for cancer stem cells in lung cancer, and immunohistochemical staining revealed that ALDH1A1, but not ALDH3A1, was significantly correlated with prognosis 8. However, ALDH3A1 expression associated with tumor progression and metastasis has extensively been reported. In human lung adenocarcinoma cisplatin resistance cell line, ALDH3A1 was screened and might participate in tumor resistance 9. In prostate cancer, ALDH3A1 was expressed in cancer stem cells related to drug-resistance 10. In pancreatic cancer, ALDH3A1 was a critical gene that affected cancer cell proliferation and drug resistance 11. Therefore, ALDH3A1 was involved in tumor progression, but ALDH3A1 expression in metastasis LUAD and its associated mechanism remain poorly understood.

Bioinformatics analysis has recently been utilized for the profiling of tumor tissues. In our previous study, differently expressed genes were analyzed between LUAD patients with metastasis (M1) and without metastasis (M0) 12. To further analyze these differently expressed genes, ALDH3A1 was significantly upregulated in metastasis LUAD patients. We reasoned that ALDH3A1 might be a crucial regulator of progression and recurrence in LUAD patients.

## Materials and Methods

### Human specimens

46 lung cancer patients diagnosed with LUAD were enrolled from the first affiliated hospital of Zhengzhou University. The institutional research ethics committee has approved this study, and all patients provided informed consent. Enrolled LUAD patients did not receive any anti-tumor therapy before surgery, and tumor tissues were obtained. And clinical information was showed in Table 1.

mRNA-seq data in LUAD patients was collected from The Cancer Genome Atlas (TCGA). The screening criteria and differently expressed genes analysis between M0 and M1 were demonstrated in a previous study 12.

### Cell culture

The lung cancer cell lines (A549 and Calu3), BEAS-2B, and 293T cell lines were purchased from Shanghai Cell Bank, Chinese Academy of Sciences. A549 and Calu3 were cultured by Dulbecco's modified Eagle's medium (DMEM) (Sigma, USA) containing 10% FBS, 10 units/mL penicillin and 10 µg/mL streptomycin. Roswell Park Memorial Institute (RPMI) 1640 media (Sigma, USA) supplemented with 10% FBS, 10 units/mL penicillin, and 10 µg/mL streptomycin were utilized to culture BEAS-2B at 37 °C in a 5% CO_2_ incubator. 293T cell line was cultured with DMEM high-glucose medium (Sigma, USA) including 10% FBS, 10 units/mL penicillin, and 10 µg/mL streptomycin.

### Small hairpin RNA (shRNA) design and retroviral gene transduction

The lentivirus plasmids with shRNA sequences were purchased from GenePharma (Shanghai, China). ALDH3A1-sh1, ALDH3A1-sh2, BAG1-sh and control-sh vector were transfected into 293T cells with three packaging plasmids using Lipofectamine 3000 (Invitrogen, USA) according to the direction. The supernatants with lentivirus were collected at 72 hours to transduced A549 cell lines. A549 infected with shRNAs were further cultured to determine the transfection efficiency.

### RNA isolation, Reverse Transcription-Polymerase Chain Reaction (RT-PCR), and Real-Time PCR

Total RNAs from tumor tissues and cell lines were isolated using TRIzol reagent (TAKARA, Japan). According to kit instructions, one microgram RNA per sample was utilized as the template for reverse transcription utilizing PrimeScript™ RT reagent Kit with gDNA Eraser (TAKARA, Japan). Briefly, gDNA was firstly removed by gDNA eraser at 42 °C for 5 minutes. Secondly, RNAs with RT primer mix and PrimeScript RT enzyme mix I were reacted to acquire cDNAs in PrimeScript buffer for 15 minutes.

For real-time PCR, 2 µL cDNA was used as a template with 1 μM forward primer, 1 μM reverse primer, and 5 μL FastStart Essential DNA Green Master reagent mix (Roche, USA). The reaction system was shown as follows: 95 °C for 10 minutes, followed by 40 cycles of 95 °C for 10 seconds, 65 °C for 10 seconds, and 72 °C for 10 seconds. 2^-ΔΔCt^ was employed to calculate gene relative expression, and β-actin was deployed as an internal control gene.

### Western Blot

Culture cell lines were lysed to acquire protein for gene expression detection. Firstly, sodium dodecyl sulfate-polyacrylamide gel electrophoresis (SDS-PAGE) was used to separate the target proteins and further transferred to nitrocellulose blotting membranes (GE healthcare life science, Germany). Next, the membranes were blockaded by 10% skim milk powder, followed by incubation with the following primary antibodies overnight at 4 °C: β-actin (8H10D10) Mouse mAb #3700 (CST, USA), p53 (DO-7) Mouse mAb #48818 (CST, USA), Bag1 (3.10G3E2) Mouse mAb #3920 (CST, USA), and anti-ALDH3A1 antibody (ab186726) (Abcam, UK). On the next day, the membranes were incubated with anti-mouse IgG, HRP-linked Antibody #7076 (CST, USA) and anti-rabbit IgG, HRP-linked Antibody #7074 (CST, USA) at room temperature after washing three times with phosphate buffer solution (PBS) containing 0.05% Tween20. Finally, the proteins' expression was detected by Pierce™ ECL Western Blotting Substrate (Thermo Fisher Scientific, USA).

### Cell Counting Kit-8 (CCK-8) assay

Tumor cell lines' proliferation was evaluated using CCK-8 (HY-K0301) (MedChemExpress, USA). 5 × 10^3^ tumor cells per well were seeded in a 96-well plate with 100 µL culture medium in a 5% CO_2_ incubator at 37 °C. CCK-8 solution (10 µL) was directly added into each well after 24, 48 and 72 hours. The plate was incubated for one hour, and absorbance per well was measured at 450 nm using an automatic microplate reader (Thermo Fisher Scientific, USA).

### Wound healing assay

Tumor cell migration was detected by wound healing assay. A total of 3 × 10^5^ cells were seeded into a 24-well plate and cultured overnight. Then, a sterile silicone insert was used to building the cell-free gap. After cultured for 24 and 48 hours, tumor cells were photographed under a microscope. Finally, the gap proportion covered by cells at each time was calculated to evaluate the migration capacity.

### Invasion assay

A 6.5 mm transwell with 8.0 μm pore polycarbonate membrane insert (Corning, USA) was utilized in invasion assay. Firstly, 100 μL diluted growth factor reduced matrigel basement matrix (Corning, USA) was added into transwell inserts for one hour at 37 °C. A 100 µL 3 × 10^5^/mL tumor cell suspension was seeded into upper chamber coated matrigel in serum-free DMEM medium, and 600 µL DMEM containing 10% FBS medium was added into lower chamber. After that, cells' suspension in upper chamber was removed and transwell membrane was gently wiped with a moistened cotton swab to remove the matrigel and cells. Next, 4% paraformaldehyde was used to fix the cells in transwell membrane for 30 minutes, and 1% crystal violet was used to stain membrane for 5 minutes. After washing three times, transwell membrane was dried and photographed under a microscope to count cell number.

### Cell metabolism detection

Cell glycolysis was measured by extracellular acidification rate (ECAR) using XF Glycolysis Stress Test Kit (Agilent, USA) according to kit instructions. Briefly, the sensor cartridge was hydrated in Seahorse XF Calibrant at 37 °C in a CO_2_-free incubator overnight. On the next day, 180 µL 2 × 10^5^/mL tumor cell suspension was added into a 96-well cell culture plate in Seahorse XF Base Medium containing 1 mM glutamine and 20 µL diluted glucose, while 20 μL diluted oligomycin, and 25 μL diluted 2-DG were added into sensor cartridge. Finally, the seahorse XF glycolysis stress test was run by wave software (Agilent, USA) and was used to automatically calculate ECAR. Besides, the concentration of glucose and lactate in tumor cell was also detected by glucose assay kit (ab65333) (Abcam, UK) and lactate assay kit (K607-100) (Biovision, USA) according to manufacturer instructions, respectively.

### Flow cytometry

Tumor cells in culture plate were digested to acquire single-cell suspension, which was washed by PBS three times. APC anti-human Ki-67 (Biolegend, USA) and 7-AAD viability staining solution (Biolegend, USA) were added into cell suspension for 30 minutes at 4 °C. Fluorescence intensity was measured using a flow cytometer (BD Biosciences, USA). Finally, data were analyzed by FlowJo V10 software.

### Immunohistochemistry (IHC)

Human lung cancer tissues were fixed and paraffin-embedded. The specimens were incubated at 65 °C for 1 hour and dewaxed in dimethylbenzene and different alcohol concentration gradients. Paraffin-embedded slides were incubated with primary antibodies of ALDH3A1 (ab186726) (Abcam, UK) and BAG1 (ab32109) (Abcam, UK) overnight at 4 °C after antigen retrieval. On the next day, paraffin-embedded slides were incubated with the secondary antibody. 3, 3-diaminobenzidine tetrahydrochloride (DAB) was used to stain target proteins, and immunostaining per slide was assessed by positive tumor cells.

### Statistical Analysis

All *in vitro* experiments were independently performed at least three times, and data were expressed as mean ± standard error of mean (SEM). Student's *t*-test, one-way ANOVA test and Pearson correlation analysis were employed for statistical analysis, and *p*<0.05 was considered significant. The graphs were depicted using GraphPad Prism 8 and Adobe Illustrator 2020.

## Results

### ALDH3A1 is positively associated with clinical poor prognosis

**Figure 1:**
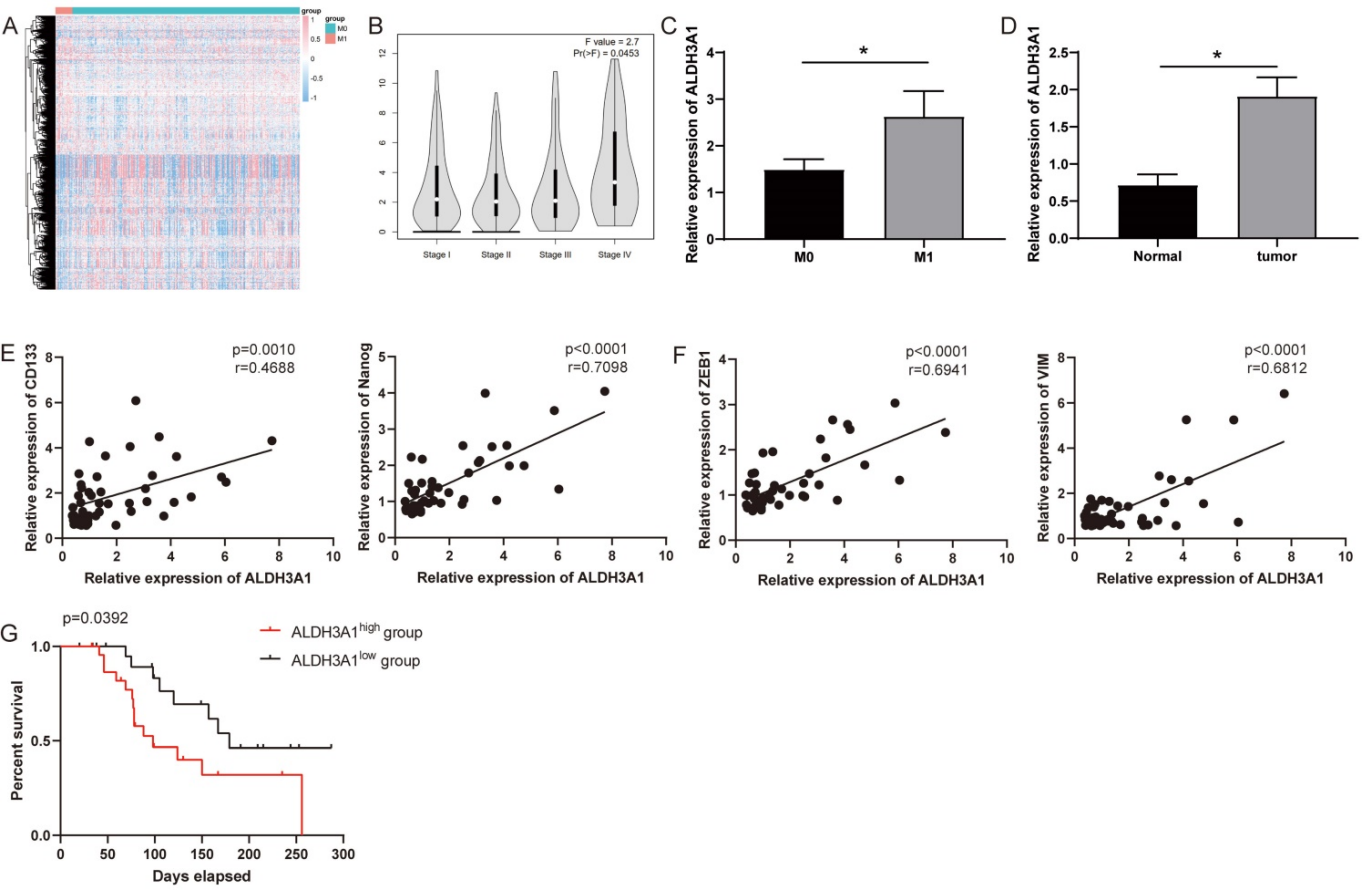
** ALDH3A1 influences tumor progression and prognosis in LUAD.** (A) Differently expressed genes were screened in M0 and M1 patients from TCGA. (B) ALDH3A1 expression in different tumor stages in GEPIA. (C) ALDH3A1 expression in M0 and M1 patients in enrolled LUDA patients. And (D) ALDH3A1 expression in normal and tumor tissues. Correlation analysis between ALDH3A1 and cancer stem cell-associated genes (E) or EMT-associated genes (F). (G) Kaplan-Meier survival curve of enrolled LUAD patients with high or low expression of ALDH3A1 within tumor specimens. X-axis represents the survival time, and Y-axis represents the survival ratio of patients.

Striving toward elucidating key genes that regulate metastasis, we analyzed mRNA sequencing data collected from TCGA in 21 tumor tissues from LUAD patients with M1 versus 283 tumor tissues from LUAD patients with M0. Differential expression analysis was performed and generated heat-map for 981 differentially expressed genes (Figure 1A). ALDH3A1 was the top one and most significantly upregulated gene (Supplementary Table 1). It was identified using Gene Expression Profiling Interactive Analysis (GEPIA) that ALDH3A1 expression in later-stage tumor tissues was significantly higher than that in early-stage tumor tissues in LUAD patients (Figure 1B). We enrolled 46 LUAD patient samples for gene detection and found that ALDH3A1 expression was higher in M1 patients than that in M0 patients (Figure 1C). Moreover, ALDH3A1 also significantly high expressed in tumor tissue compared to normal tissue (Figure 1D). A large body of evidence suggested that epithelial-mesenchymal transition (EMT) and cancer stem cells were the major factors contributing to tumor metastasis 13-15. ALDH3A1 expression increased significantly with increasing stem cell-associated genes' expression (CD133 and Nanog) (Figure 1E) and EMT-associated genes' expression (ZEB1 and VIM) (Figure 1F). Survival was analyzed according to low ALDH3A1 mRNA versus high ALDH3A1 mRNA. Kaplan-Meier plots revealed a significant difference in survival time between low and high ALDH3A1 mRNA groups (Figure 1G). These data suggested that ALDH3A1 exhibited a relatively high correlation with metastasis and might serve as a biomarker for tumor metastasis in LUAD.

### ALDH3A1 promotes lung cancer cell proliferation

**Figure 2:**
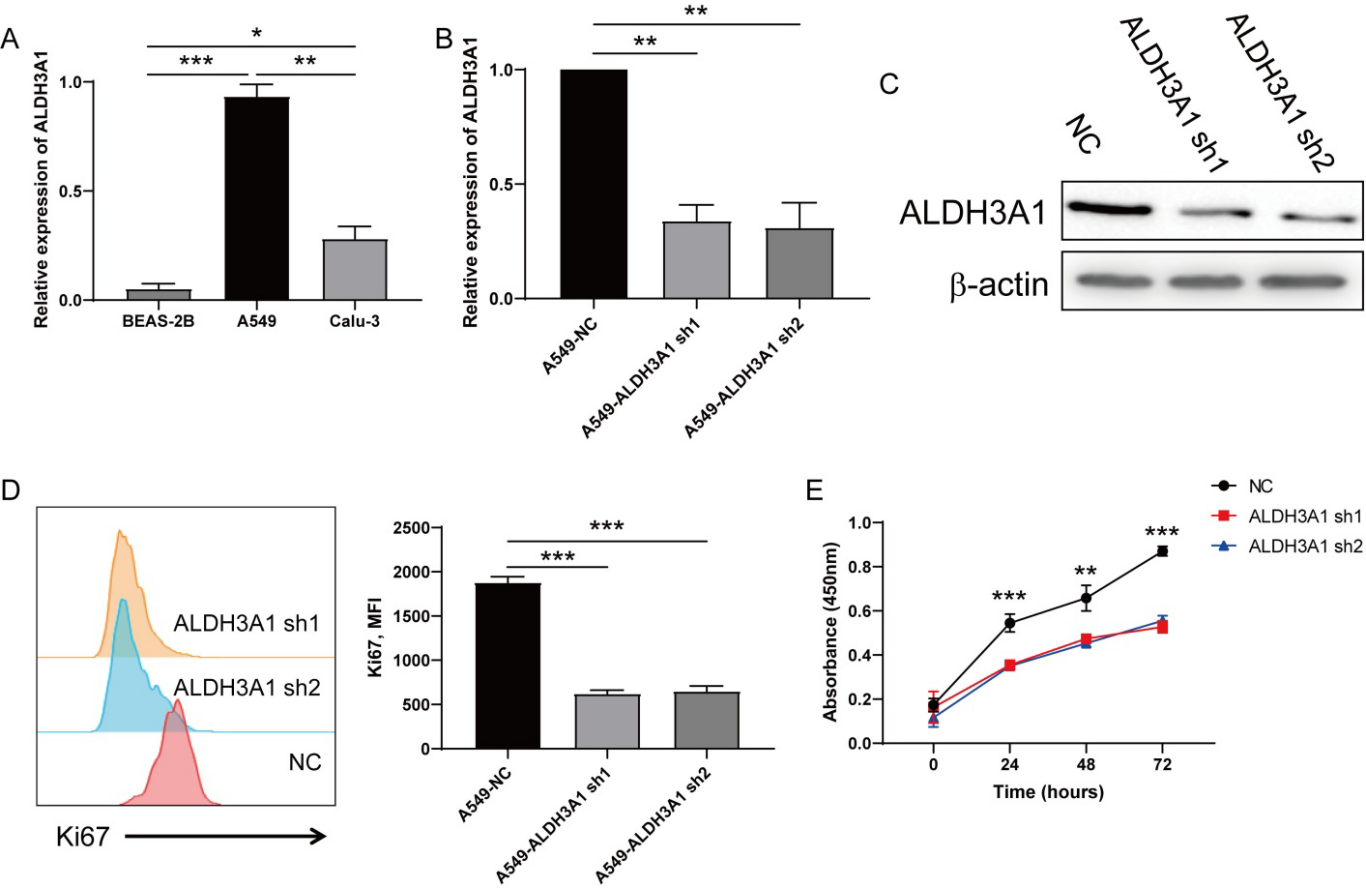
** Downregulation of ALDH3A1 inhibits proliferation in lung cancer cells.** (A) Expression of ALDH3A1 in normal lung cell line BEAS-2B and lung cancer cell lines A549 and Calu3. Knockdown efficiency of ALDH3A1 in A549 cell line transduced with ALDH3A1-sh1 and ALDH3A1-sh2 by real-time PCR (B) and western blot (C). (D) Mean fluorescence intensity (MFI) of Ki67 in A549 cell line transduced with ALDH3A1-sh1 and ALDH3A1-sh2 was detected by flow cytometry. (E) Tumor cell growth from A549 cell line transduced with ALDH3A1-sh1 and ALDH3A1-sh2 by CCK-8, and absorbance per well was measured at 450 nm using an automatic microplate reader. Data were expressed as mean ± SEM. *p < 0.05, **p < 0.01, ***p < 0.001.

To further investigate ALDH3A1 role, ALDH3A1 expression was detected in LUAD cancer cell lines (A549 and Calu3) and BEAS-2B. This result exhibited that ALDH3A1 was higher expressed in LUAD cancer cell lines than normal lung cell lines, and the A549 cell line showed the highest expression of ALDH3A1 (Figure 2A). Vectors of negative control (NC)-sh, ALDH3A1-sh1 and ALDH3A1-sh2 were constructed to analyze the direct regulatory capability of ALDH3A1 for tumor metastasis. The knockdown efficiency was confirmed by real-time PCR (Figure 2B) and western blot assay (Figure 2C). Besides, following ALDH3A1 knockdown, a rapid decline of Ki67 expression (Figure 2D) suggested that ALDH3A1 might regulate cancer cells proliferation. Moreover, CCK-8 assay was performed, and A549 cell lines infected with ALDH3A1-sh1 and ALDH3A1-sh2 exhibited a lower proliferation rate than NC cell line at 24, 48 and 72 hours (Figure 2E).

### ALDH3A1 facilitates migration and invasion of lung cancer cell

**Figure 3:**
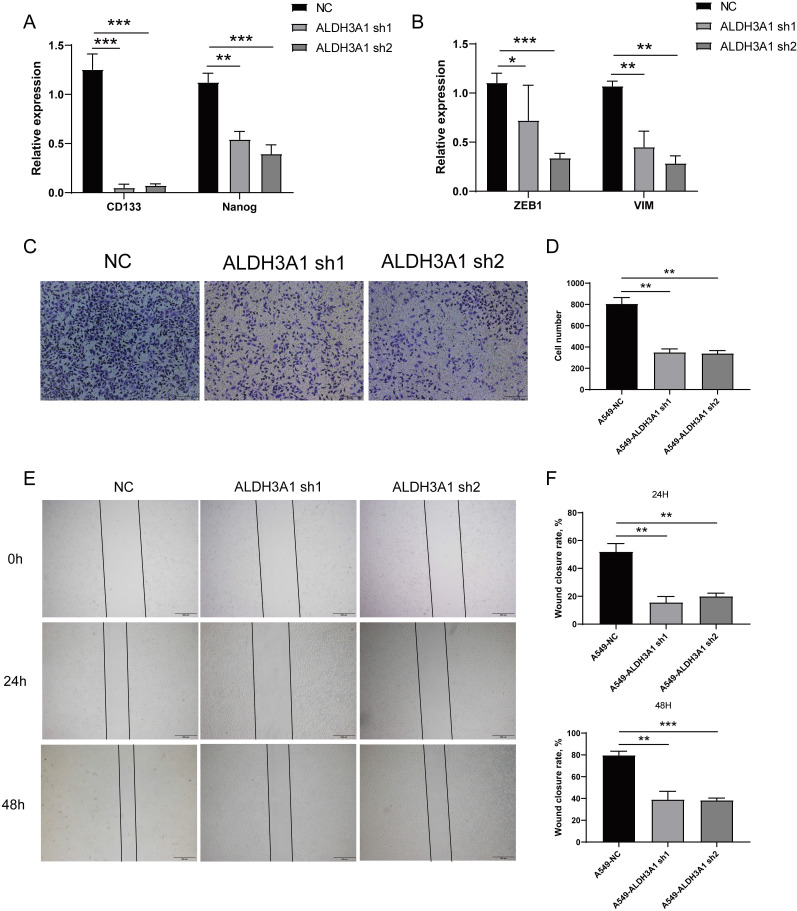
** ALDH3A1 induces migration and invasion in tumor cell lines.** Cancer stem cell-associated genes (A) or EMT-associated genes (B) expression in A549 cell line transduced with ALDH3A1-sh1 and ALDH3A1-sh2. Representative images of invasion (C) and statistical analysis of invasion cell numbers per field (D) in A549 cell line transduced with ALDH3A1-sh1 and ALDH3A1-sh2. Representative images of migration (E) and statistical analysis (F) in A549 cell line transduced with ALDH3A1-sh1 and ALDH3A1-sh2. Data were expressed as mean ± SEM. *p < 0.05, **p < 0.01, ***p < 0.001.

As shown in Figures 1C and D, ALDH3A1 showed a positive correlation with cancer stem cell and EMT. We further identify whether ALDH3A1 affected these features. CD133 and Nanog down-regulated in A549 cell lines with ALDH3A1-sh1 and ALDH3A1-sh2 (Figure 3A). Besides, EMT-associated genes exhibited the same trends in knockdown cell lines (Figure 3B). Since the invasion and migration ability are two important biological characteristics of metastasis, Trans-well and wound healing assays were performed. The numbers of invaded cells (Figure 3C and D) and wound closure rate (Figure 3E and F) in A549 cells with ALDH3A1-sh1 and ALDH3A1-sh2 were significantly decreased. Overall, it can be concluded that ALDH3A1 could remarkably promote migration and invasion of LUAD cells.

### Cell metabolism of lung cancer is increased by ALDH3A1

**Figure 4:**
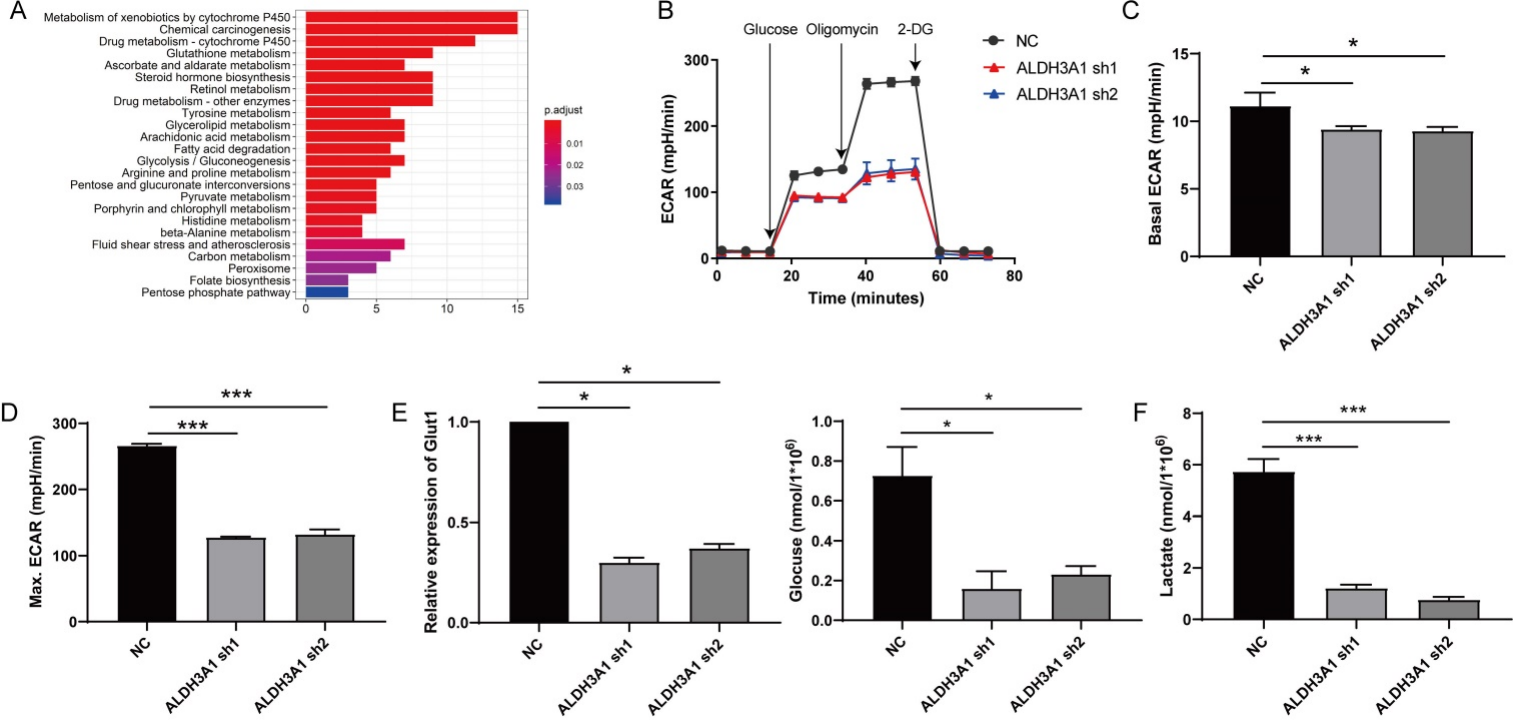
** Tumor cell with low expression of ALDH3A1 exhibits poor glycolysis.** (A) Different pathway analysis between low and high ALDH3A1 mRNA groups. (B) Seahorse assay for ECAR detection in A549 cell line transduced with ALDH3A1-sh1 and ALDH3A1-sh2. Basal ECAR (C), maximum ECAR (D), Glut1 expression, glucose consumption, (E) and lactate production (F) in NC, ALDH3A1-sh1 and ALDH3A1-sh2 cell lines. Data were expressed as mean ± SEM. *p < 0.05, ***p < 0.001.

RNA sequence data of LUAD patients from TCGA was also used to perform Kyoto Encyclopedia of Genes and Genomes (KEGG) pathway analysis between low and high ALDH3A1 mRNA groups. This result revealed that LUAD patients with high ALDH3A1 expression displayed strong transcriptional signatures of metabolism, such as glycolysis, pyruvate metabolism, carbon metabolism and fatty acid metabolism (Figure 4A). Enhanced glycolysis in cancer cells would be predicted to promote tumor cell growth and metabolism in various cancers, such as colorectal cancer, pancreatic cancer, and so on 16-18. To verify whether ALDH3A1 might regulate glycolysis, ECAR was performed. Decreased ALDH3A1 expression had a major impact on ECAR rate (Figure 4B), suggesting that ALDH3A1 was critical for tumor metastasis. ALDH3A1 expression maintained basal ECAR rate in tumor cell (Figure 4C). Treatment with oligomycin might enlarge tumor cell ECAR, and we stated that ALDH3A1 still positively affected ECAR rate (Figure 4D). Besides, glucose absorption and lactate production are major glycolysis characters. Glut1 was down-regulated after AlDH3A1 was knocked down (Figure 4E). A549 cell lines infected with ALDH3A1-sh1 and ALDH3A2 exhibited lower glucose absorption (Figure 4E) and lactate production (Figure 4F). Therefore, ALDH3A1 might be dependent on glycolysis to maintain tumor cell metastasis.

### ALDH3A1 regulates cell proliferation through p53/BAG1 in lung cancer cell line

**Figure 5:**
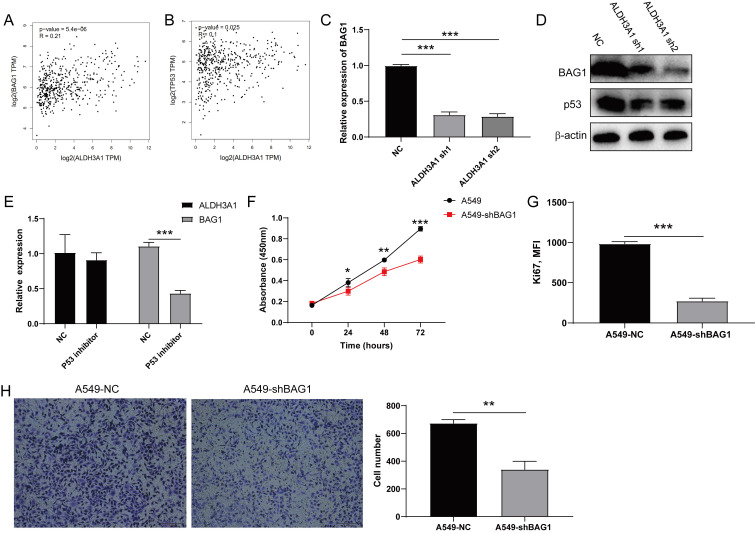
** ALDH3A1 regulates tumor cell proliferation through p53/BAG1 axis.** The correlation between ALDH3A1 and BAG1 (A) or TP53 (B) in GEPIA. (C) BAG1 expression in NC, ALDH3A1-sh1 and ALDH3A1-sh2 cell lines. (D) The expression of BAG1, p53 and β-actin detected by western blot in NC, ALDH3A1-sh1 and ALDH3A1-sh2 cell lines. (E) ALDH3A1 and BAG1 expression in tumor cell lines treated with p53 inhibitor. (F) Tumor cell growth from A549 cell line transduced with BAG1-sh by CCK-8, and absorbance per well was measured at 450 nm using an automatic microplate reader. (G) MFI of Ki67 expression detected by flow cytometry in tumor cell transduced with BAG1-sh. (H) Representative images of invasion and statistical analysis of invasion cell numbers per field in A549 cell line transduced with BAG1-sh. Data were expressed as mean ± SEM. *p < 0.05, **p < 0.01, ***p < 0.001.

To explore the target gene of ALDH3A1, we acquired 200 genes that were positively correlated with ALDH3A1 after analyzing RNA data of LUAD patients in TCGA (Supplementary Table 2). BAG1 ranked in the top 5 genes was extensively reported and could increase tumor cell proliferation and decrease apoptosis 19, 20. Moreover, the correlation analysis in GEPIA also stated that BAG1 exhibited a significant relationship with ALDH3A1 (Figure 5A). Besides, ALDH3A1 expressing cell lines showed a higher TP53 expression 21, and we found that TP53 expression was upregulated in LUAD patient tissues from GEPIA with high ALDH3A1 expression (Figure 5B). Therefore, we hypothesized that BAG1 might be a target gene of ALDH3A1 by regulating p53. Real-time PCR assay and western blot revealed that BAG1 expression was decreased in A549 cell lines with ALDH3A1-sh1 and ALDH3A1-sh2 (Figure 5C and D), which also showed lower p53 expression by western blot. To better prove our speculation, treatment with TP53 inhibitor Pifithrin-α (PFTα) successfully decreased BAG1 expression but did not affect ALDH3A1 expression (Figure 5E), demonstrating that BAG1 was the downstream element of TP53. A549 cell line infected with sh-BAG1 lentivirus exhibited a lower proliferation capacity (Figure 5F and G) and higher invasion ability (Figure 5H) than the control group. Overall, our analysis revealed that ALDH3A1 mainly maintained tumor cell metastasis by p53/BAG1 axis.

### BAG1 regulated by ALDH3A1 predicts clinical poor prognosis

**Figure 6:**
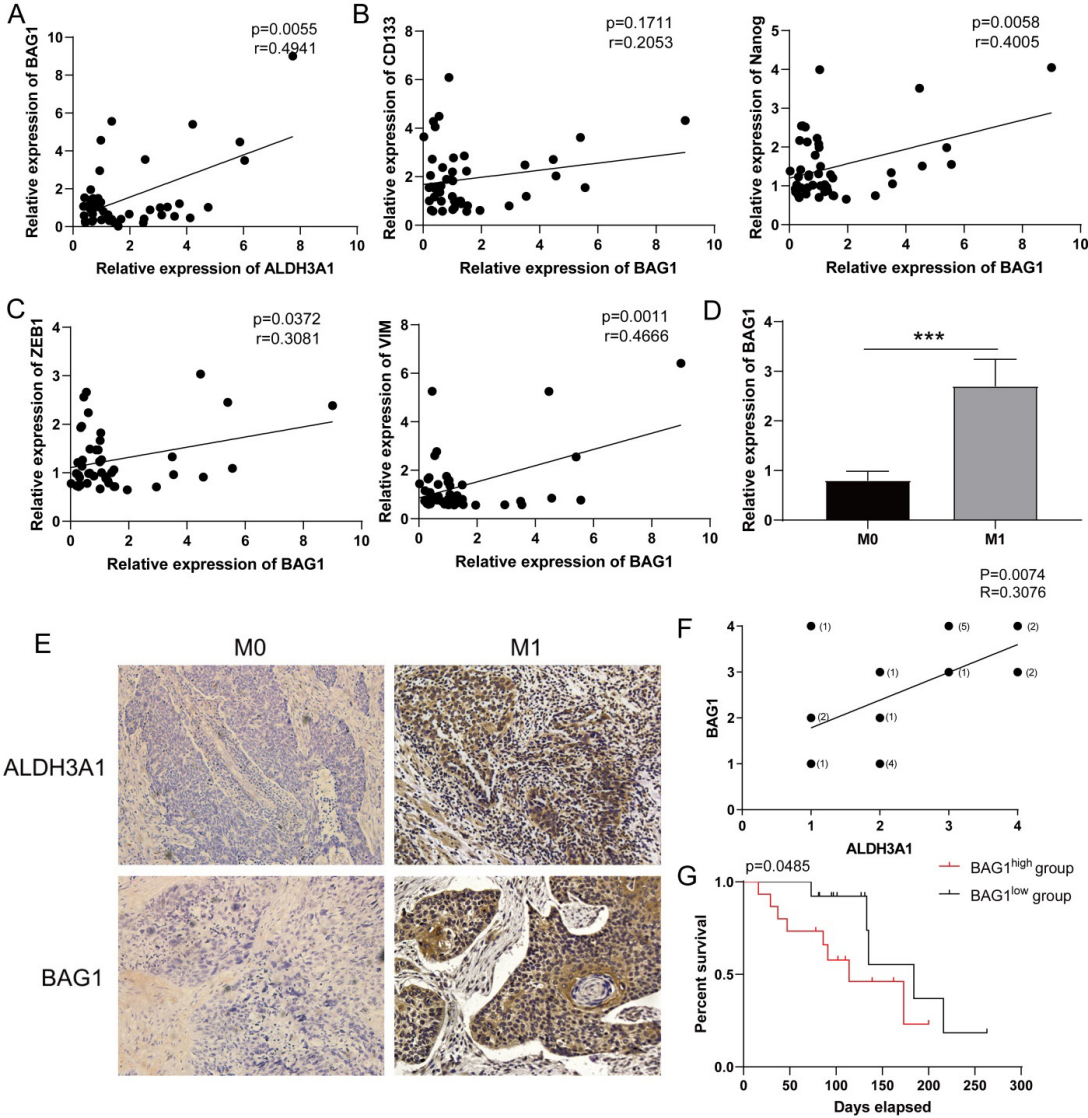
** Low expression of BAG1 is associated with good prognosis.** (A) Correlation analysis of BAG1 and ALDH3A1 in enrolled LUAD patients. Correlation analysis between BAG1 and cancer stem cell-associated genes (B) or EMT-associated genes (C). (D) BAG1 expression in M0 and M1 LUAD patients. (E) IHC staining for ALDH3A1 and BAG1 in LUAD cancer tissues and (F) correlation analysis between BAG1 and ALDH3A1. (G) Kaplan-Meier survival curve of enrolled LUAD patients with high or low expression of BAG1 within tumor specimens. X-axis represents the survival time, and Y-axis represents the survival ratio of patients. Data were expressed as mean ± SEM. *p < 0.05, **p < 0.01.

Consistent with the results in Figure 5A, the relationship between ALDH3A1 and BAG1 was identified in enrolled LUAD patient samples (Figure 6A). BAG1 expression was positively associated with stem cell-associated genes' expression (CD133 and Nanog) (Figure 6B) and EMT-associated genes' expression (ZEB1 and VIM) (Figure 6C). Moreover, such patients were divided into M0 group and M1 group. BAG1 presented a significantly higher expression in M1 group than that in M0 group (Figure 6D). We next found BAG1 exhibited a positive correlation with ALDH3A1 (Figure 6E and F). According to BAG1 protein level, LUAD patients were segmented in BAG1 high expression and low expression. Survival analysis revealed that survival time in BAG1 low expression group was elevated (Figure 6G). These results indicated that ALDH3A1 promoted LUAD metastasis and progression through modulating BAG1.

## Discussion

Although metastasis is a known phenomenon in LUAD, information is limited about regulatory factors and underlying mechanisms of LUAD development and progression. Herein, we demonstrated for the first time that ALDH3A1 caused LUAD cell proliferation, migration, invasion, and metabolism by p53/BAG1 axis for cancer metastasis. LUAD cell lines transduced with ALDH3A1-sh substantially reduced the tumor cell proliferation, migration, and invasion, suggesting that ALDH3A1 contributes to potential development of LUAD. High-expression ALDH3A1 induces high ECAR, rapid glucose consumption and lactate accumulation. The progression in LUAD patients was predominantly associated with high expression of ALDH3A1 and BAG1.

Consistent with preceding studies, we demonstrated that ALDH3A1 displayed a high expression level in metastasis colon cancer cells compared with primary colon cancer cells and provided potential biomarkers to develop new therapy to target stem cell caused metastasis 22. ALDH3A1 was also screened in gastric adenocarcinoma to be used as the prognosis marker 23. Although ALDH3A1 was screened and presented a predictive role for tumor progression in different cancers, few studies identified this phenomenon. Our study demonstrated that ALDH3A1 in LUAD tumor samples of patients with M1 was significantly upregulated than in patients with M0. Accumulating evidence demonstrated that ALDH3A1 could influence tumor cell proliferation 23-26. We, therefore, explored the potential role of ALDH3A1 in mediating cancer cell proliferation. Along with sequentially increased ALDH3A1 in LUAD cells, the migration capacity, invasion, and metastasis increased along with cell proliferation. This was consistent with the report that ALDH3A1 carried by exosomes strongly enhanced tumor cell migration and invasion by accelerating glycolysis 27.

To identify the molecular mechanism of ALDH3A1 in LUAD patients, we observed that p53 regulated by ALDH3A1 could influence BAG1 expression that was reported for the first time. A study reported another mechanism that IL-6/STAT3 pathway could be activated through ALDH3A1, contributing to reduced cell proliferation, migration, and invasion in oral squamous cell carcinoma 28, which was in consistent with our findings. However, several studies confirmed that ALDH3A1 was positively associated with tumor cells' progression, which was found in our research 29-32. Also, BAG1 highly expressed in gallbladder carcinoma samples was inhibited by microRNA-138, increasing tumor cell proliferation 33. In gastric cancer, BAG1 expression could be downregulated by microRNA-494, leading to decreased cell proliferation and enhanced apoptosis 34. Our study found another mechanism that BAG1 expression was induced by ALDH3A1/p53, which provided a new insight for tumor metastasis research. Previous reports have demonstrated increased prevalence of BAG1 in breast cancer and clear-cell renal cell carcinoma 35-37, supporting our observation in this study. Enhanced BAG1 expression was associated with a better prognosis in metastatic renal cell carcinoma 38, which was inconsistent with our LUAD patients' results. However, BAG1 was found to participate in breast cancer progression and drug resistance and showed a positive association with survival time in breast cancer 39, 40. These results support our observations and further indicate that inhibiting BAG1 might represent a means that prevent tumor progression in LUAD patients. Our findings provide new insights into the potential roles of ALDH3A1/p53/BAG1 axis in LUAD progression.

## Conclusion

Overall, our work highlights that ALDH3A1 is highly expressed in M1 LUAD patients and associated with cancer stem cell property. Furthermore, ALDH3A1 down-regulation in human LUAD may affect tumor cell proliferation, migration, and invasion. In addition, ALDH3A1 also contributes to cell metabolism, such as glycolysis, pyruvate metabolism, carbon metabolism and fatty acid metabolism. Moreover, the underlying mechanism is uncovered that ALDH3A1 influences tumor cell metastasis through p53/BAG1 axis. Collectively, our findings shed light on ALDH3A1 importance for maintaining cancer cell metastasis in LUAD, providing a promising and novel factor for lung cancer treatment.

## Supplementary Material

Supplementary table 1.

Supplementary table 2.

## Figures and Tables

**Table 1:** Clinical characteristics in LUAD patients

Parameters	Number	Mean value (ALDH3A1 expression)	*P*
**Age (years)**			0.6278
<60	24	1.623	
≥60	22	2.226	
**Gender**			0.6597
Male	30	1.875	
Female	16	2.125	
**Metastasis**			0.0005
M0	32	0.806	
M1	14	2.706	
**Clinical stage**			0.6899
I	34	1.983	
II-III	12	1.763	

## Abbreviations

LUAD: lung adenocarcinoma
ALDH3A1: aldehyde dehydrogenase 3A1
shRNA: Small hairpin RNA
RT-PCR: reverse transcription-polymerase chain reaction
CCK-8: Cell Counting Kit-8
TCGA: The Cancer Genome Atlas
GEPIA: Gene Expression Profiling Interactive Analysis
EMT: epithelial-mesenchymal transition
